# Visual information modulates brain network characteristics during static balance following ACL reconstruction – A graph theoretical analysis

**DOI:** 10.1038/s41598-026-52086-6

**Published:** 2026-05-06

**Authors:** Adam Grinberg, Tim Lehmann, Johan Strandberg, Gjergji Cobani, Charlotte K. Häger

**Affiliations:** 1 Department of Community Medicine and Rehabilitation, Umeå University, Umeå, Sweden; 2 Exercise Science & Neuroscience Unit, Department of Exercise & Health, Faculty of Science, Paderborn University, Paderborn, Germany; 3 Department of Statistics, Umeå University, Umeå, Sweden; 4 Department of Diagnostics and Intervention, Umeå University, Umeå, Sweden; 5 Clinics of Orthopaedics, University Hospital of Umeå, Umeå, Sweden

**Keywords:** Anterior cruciate ligament, postural control, EEG, functional connectivity, graph theory, brain network segregation, Motor control, Rehabilitation

## Abstract

Long-term balance impairments are prevalent after anterior cruciate ligament (ACL) injury and are possibly linked to an overreliance on visual information and related cortical processing. We therefore aimed to explore characteristics of functional brain networks related to postural control with and without vision following ACL reconstruction (ACLR). Twenty-seven individuals after ACLR and 24 non-injured controls performed single-leg balance tasks under eyes-open/eyes-closed conditions. Graph-theoretical measures of functional network segregation (clustering coefficient, CC) and integration (path length, PL) were derived from mobile electroencephalography. Sway characteristics were calculated based on centre of pressure (CoP; area and velocity) and the mean distance between CoP and centre of mass (CoM). Knee antero-posterior kinematics were also explored. Group effects were analysed using permutation-based ANCOVA. During eyes-open only, the ACLR group exhibited greater cortical network segregation (higher CC; *p* = 0.025) in the alpha-1 band (8–10 Hz). While sway characteristics were similar between groups, the ACLR leg demonstrated greater knee flexion compared to their contralateral leg (*p* = 0.036). Individuals post-ACLR showed more efficient functional brain connectivity during eyes-open, combined with kinematic adaptations in their injured leg. These findings suggest post-ACLR neural adaptations of postural control mechanisms, particularly when visual information is available.

**Supplementary Information:**

The online version contains supplementary material available at 10.1038/s41598-026-52086-6.

## Introduction

Anterior cruciate ligament (ACL) injuries are common in team sports,^1^ often leading to long-term alterations in functional movements, that may persist many years after the injury.^2–5^ Apart from mechanical deficiencies, impaired proprioception has been widely reported in various stages following an injury^6^ and is believed to be subsequent to initial loss of ACL mechanoreceptors.^7,8^ The integration of proprioceptive, visual, and vestibular information by the central nervous system is crucial for human postural control, a set of neural processes that monitor body position and alignment in space.^9^ Consequently, impaired proprioception and less efficient integration of constantly changing sensory information may inherently lead to postural instability. Indeed, the ability to maintain a simple single-leg stance with eyes open has been shown to be deficient after ACL injury.^10^ These impairments in static balance, reflected by increased sway area and velocity, have been observed even two years after ACL-reconstruction (ACLR) for the injured leg compared with the contralateral leg,^11^ as well as compared with non-injured controls.^12^ A long-term investigation^5^ further confirmed that single-leg balance remains inferior at more than 20 years following ACL-injury regardless of whether surgical reconstruction was included in the treatment, and interestingly also for the non-affected leg, which supports central nervous system involvement. Investigations utilising brain imaging methods, such as functional magnetic resonance imaging (fMRI) or electroencephalography (EEG) suggest that such long-term deficits may originate in alterations of somatosensory information from the knee and are further compounded by subsequent neural adaptations in cortical processing.^13–15^ In this regard, individuals following ACLR exhibit reduced innervation to the primary sensory cortex,^16^ and show variations in corticospinal and motor cortex excitability.^15,17,18^ Together, these findings suggest changes in sensorimotor integration and corticospinal responsiveness. Thus, to achieve effective control of knee motion and stability, particularly in the injured leg, individuals may require greater cortico-cortical stimulation to compensate for these altered excitability patterns. While preliminary observations have already identified compensatory cortical patterns associated with proprioceptive tasks following ACL reconstruction,^19–21^ critical gap remains with regards to the characteristics of compensatory cortical mechanisms related to postural deficiencies in this population. Previous work^22^ has already indicated that individuals two years after ACLR exhibit distinct compensatory cortical activation patterns during single leg standing, with greater activation of the somatosensory and visual cortices compared to non-injured individuals, despite similar postural sway characteristics. In line with this, stronger statistical interdependencies (functional connectivity) within a distributed network of sensorimotor areas were observed in individuals after ACLR, predominantly encompassing fronto-parietal and occipito-motor regions of the brain.^23^ These two initial studies concordantly indicate increased demands on the postural control system in individuals with ACLR to maintain postural equilibrium. Distinctions in occipito-motor and parieto-motor functional connections further support a tendency towards an increased dependence on visual processing in ACLR individuals.^14^ Such alterations of functional connectivity patterns may reflect compensatory adaptations in task-related global and local brain networks following ACLR, placing greater emphasis on active cortical processing as postural control becomes less automatic.^24^ In this context, graph theory provides a powerful framework for modelling pairwise interactions within large-scale brain networks,^24,25^ thus offering a quantifiable approach to mapping neurophysiological processes of postural control under various conditions.^26^ EEG-derived brain graphs are composed of individual electrodes or cortical regions, represented as nodes, and functional connections between them as edges. By computing the local interconnectedness of a network’s nodes (segregation) and the efficiency of information transfer within it (integration), graph theory enables to characterise the complex multivariate nature of connectomes.^24,26^ This approach offers critical insights into how sensory information influences brain network characteristics, particularly in populations with sensorimotor deficiencies.^26^

Based on the existing research, eliminating visual input may negatively affect balance performance and induce changes in the efficiency of brain network communication during a single-leg balancing task on the affected side after ACLR. However, no prior study has investigated balance performance and complementary aspects of brain network organisation after ACLR under eyes-closed conditions.

Therefore, the aim of the current study was to explore EEG-derived brain graphs describing characteristics of the functional cortical network architecture related to postural control during static one-leg standing with and without vision among individuals after ACLR. We hypothesised that individuals after ACLR would demonstrate more efficient brain network characteristics than non-injured controls in both eyes-closed and eyes-open balancing conditions, given that stronger compensatory engagement of cortical control networks might be required compared to their non-injured counterparts. Specifically, this group was expected to exhibit network configurations reflecting more local processing within specialised neighbourhoods (higher local clustering) and rapid integration of information across distant regions (shorter paths for long-range integration). If the postural task that is predominantly automatic in controls, yet attentionally controlled in individuals after ACLR, their brain networks may shift towards configurations that support global coordination for achieving similar motor outcomes as their non-injured counterparts.

## Methods

### Participants

Twenty-seven physically active individuals, following ACLR and twenty-four non-injured controls, participated in this cross-sectional investigation. Participants with ACLR were recruited mainly from the orthopaedic clinic of the regional hospital, but also from two private sports medicine clinics and through advertisements around the university campus. Controls were recruited via advertisements and convenience sampling. Inclusion criteria included 15–35 years of age, right-hand dominance,^27^ unilateral ACL injury treated with ACLR between six months to five years prior to testing, and as for physical activity level, a pre-injury Tegner activity score of 6 or above. Individuals were excluded if they had concomitant injuries other than minor meniscal tears or ligamentous sprains, provided these did not require major surgery or lead to functional deficits. Additional exclusion criteria included additional orthopaedic/neurologic pathology in the preceding year or an earlier injury with residual symptoms limiting mobility. Control participants were right-hand dominant, matched by age and physical activity level to the ACLR group and had no history of knee injury or any other musculoskeletal or neurological conditions affecting the lower limbs. All participants provided written informed consent according to the declaration of Helsinki and the study was approved by the Swedish Ethical Review Authority (Dnr 2021–03860).

### Patient-reported outcome measures (PROM)

Prior to testing, all participants filled in a set of questionnaires to provide background information regarding knee symptoms, function, physical activity level and psychological factors. The questionnaires were administered using REDCap electronic data capture tools hosted at Umeå Universtity.^28^ They included the Tegner Activity Scale (pre-injury, current; higher scores indicate higher activity level), ACL Return to Sport after Injury index (ACL-RSI; higher scores indicate better psychological readiness) and the Knee injury and Osteoarthritis Outcome Score (KOOS), which consists of five subscales (pain, symptoms, activities of daily living, sport and recreation, and quality of life), each scored from 0 to 100, with higher scores indicating better function. Participant demographics and questionnaire data are presented in Table 1.

**Table 1:** Participants’ background characteristics and questionnaire data.

		ACLR (*n* = 27)	Controls (*n* = 24)	*P*-value (Group)
Demographics & anthropometrics [mean ± SD unless otherwise stated]				
Sex (M/F)	5/22		13/11	0.010
Age (yrs)	24.0 (5.0)		24.4 (3.8)	NS
Months post-surgery, median (IQR)	17.7 (12.3–24.6)		–	–
Body height (cm)	169.9 ± 7.3		175.5 ± 9.2	0.023
Body mass (Kg)	69.6 ± 9.0		74.5 ± 13.1	NS
BMI (kg/m^2^)	24.1 ± 2.1		24.1 ± 3.1	NS
Patient-reported outcome measures [median (IQR)]				
Pre-injury activity (Tegner, 1–10)		9 (7–10)	–	–
Current activity (Tegner, 1–10)		7 (5–8)	7 (6–9)	NS
KOOS (0-100)				
Symptoms		78.6 (71.4–85.7)	96.4 (89.3–100.0)	< 0.001
Pain		91.7 (83.3–94.4)	100.0 (100.0–100.0)	< 0.001
Activities of Daily Living		100.0 (94.1–100.0)	100.0 (100.0-100.0)	NS
Sports/recreation		80.0 (65.0–90.0)	100.0 (95.0-100.0)	< 0.001
Quality of Life		62.5 (56.3–68.8)	96.9 (87.5–100.0)	< 0.001
ACL-RSI (0-100)		44.2 (31.7–61.7)	–	–

ACLR, Anterior Cruciate Ligament Reconstruction; BMI, Body Mass Index; IKDC2000, International Knee Documentation Committee Subjective Knee Form 2000; KOOS, Knee Injury and Osteoarthritis Outcome Score; ACL-RSI, Anterior Cruciate Ligament Return to Sport after Injury Survey.

### Experimental task

Single-leg measurements were conducted with participants standing barefoot on a force platform for 30 s. Two consecutive measurements were carried out, initially for the *eyes-open* condition, followed by the *eyes-closed* condition. Prior to testing, a randomisation sequence determined for each participant, whether the injured or non-injured legs would be the first to be tested. Participants performed six trials per condition, alternating between the right and left leg. Each trial started with participants standing on both legs. Upon cue, they stepped forward to the force plate area and lifted one leg, instructed to stay still as much as possible. while fixing their gaze forward towards a cross projected on a TV monitor. The non-stance leg was allowed be positioned as preferred by the participant, provided it did not touch the floor. The arms were unrestricted and could be used for balance adjustments if necessary. These adjustments were chosen to promote ecological validity and make the task more manageable, particularly in the eyes-closed condition.^29^ After 30 s, participants were cued to lower their leg and return to the starting position. In the *eye-closed* condition, if balance loss caused a participant to lower their non-stance leg, they were instructed to lift it back as quickly as possible and continue the trial as usual. This approach was implemented to preserve the ecological validity of the task and to avoid repeated restarts that could induce practice effects or fatigue. In cases where a participant had repeatedly stepped down or touched down for extended periods (more than 3 s), the trial was discarded, and an additional trial was conducted.

### Biomechanical data acquisition and processing

Kinematic data were registered at 250 Hz using an eight-camera 3D motion capture system (Oqus, Qualisys AB, Gothenburg, Sweden). Reflective passive spherical markers (*n* = 52) were attached bilaterally with double-sided adhesive tape at anatomical landmarks. Rigid clusters with four and three markers on the thighs and shanks, respectively, were used to reduce soft tissue artefacts.^30^ Markers were tracked offline in Qualisys track manager (QTM, version 2024.1, Qualisys AB, Gothenburg, Sweden). Ground reaction forces were registered at 1000 Hz using a time-synchronised triaxial force plate (Kistler Instrument AG, model 9260AA, Winterthur, Switzerland). All data were exported to Visual 3D (version 5, HAS-Motion Inc., Canada) for analysis. A fifteen-segment rigid body model consisting of head, forearm, arm, hand, trunk, pelvis, thighs, shanks and feet segments was then constructed based on marker positions. Each of the segments was given a relative mass based on Dempster’s regression Eq.^31^ (Visual 3D default segment masses) and a cylindrical geometry, for calculating the body’s centre of mass (CoM). A bi-directional second order low-pass 15 Hz Butterworth filter was then applied to both kinematic and kinetic data before further calculations of posturographic measures were performed.

### Biomechanical outcome measures

**Fig. 1:**
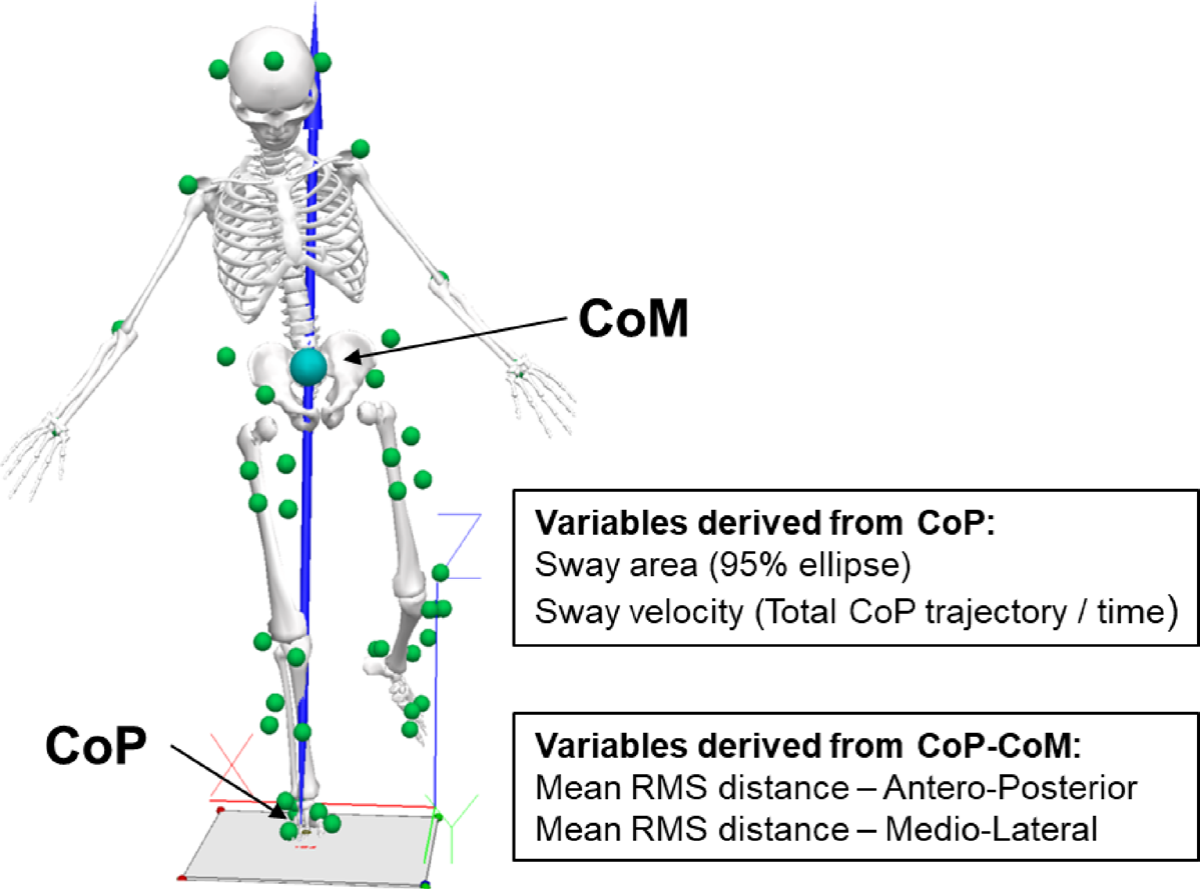
Experimental task and posturographic outcome measures. Force vector indicated by blue arrow. During 30s one-leg stance, centre of pressure (CoP) was continuously computed based on force-plate data while centre of mass (CoM) was approximated from kinematic marker-based data. Variables were derived from either the CoP or from the scalar difference between the CoP and CoM. RMS, root mean square.

For posturographic measures (Fig. 1), we extracted the dependent variables based on CoP for comparisons with previous ACLR literature,^23^ and also on the combined interpretation of the CoM and CoP. CoP-CoM represents the scalar distance between the two points at a given time,^32^ and has been proposed to reflect a more comprehensive view of postural control by incorporating the dynamical relationship between the two measures.^33,34^ CoP alone may describe sway characteristics, but doesn’t directly reflect postural instability. The CoM, however, focuses on body movement without considering the ground reaction forces that stabilise it.^32^ Therefore, variables derived from CoP-CoM offer a more accurate representation of postural control and the efficiency of the neuromuscular system in maintaining balance, as they account for both body movement (CoM) and corrective actions (CoP).^34^.

All postural parameter computations were performed in Visual 3D, based on the formulas described by Prieto et al.^35^ Variables derived from CoP included: Sway area (AREA_CoP_), calculated as the CoP 95% confidence ellipse area (mm^2^) around the sway centre, and sway velocity (MVEL_CoP_), calculated by dividing the total CoP trajectory by time (mm/s).^35^ For the calculation of CoP-CoM variables, both CoP and CoM were normalised to their respective sway centre^36^ to compensate for kinematic CoM model inaccuracies. As such, the CoP-CoM distance at each time point (i) was calculated as: *(CoP-CoM)*_*i*_ *= (CoP*_*i*_ *- CoP*_*mean*_*) – (CoM*_*i*_ *- CoM*_*mean*_*).* The root mean square (RMS) CoP-CoM distances was then calculated (mm), separately for the antero-posterior (RMS-AP_CoP-CoM_) and medio-lateral (RMS-ML_CoP-CoM_) components.^33,37^ For all the described outcome variables, higher values were interpreted as worse postural control.^33,35^.

### Complementary kinematics

While predominant balancing strategies are driven by muscle activation patterns, i.e., hip and ankle strategies,^38^ we calculated mean knee flexion angle for each trial as a kinematic measure, reflecting how participants adjusted their limb to control the CoM during the task. This provided a complementary indicator of postural adjustments.

### Touchdowns

The inclusion of eye-closed trials inevitably led to several participants losing balance, resulting in toe dips. To control for this, we implemented an automatic detection of touchdowns based on foot markers’ vertical position. Specifically, the position of three foot-markers was registered prior to foot rise for each trial. Touchdowns were initially identified based on a threshold of 1 cm between the lowest marker’s vertical position and the initial position when the foot was on the ground. Next, a manual inspection was performed of all the automatically detected touchdowns, verifying each event using the 3D kinematic data and, when needed, cross-checking with 2D video to confirm clear foot contact. False detections were then deselected. Subsequently, the number of touchdowns and relative double support time were computed, and the latter was used as a covariate in the statistical models for all outcome measures (behavioural and EEG).

### EEG recording and preprocessing

Cortical activity was continuously recorded using 65 passive Ag/AgCl electrodes (HydroCel Geodesic Sensor Net, Electrical Geodesics Inc., Oregon, USA) placed according to the extended international 10–10 system. The data were digitally amplified at a sampling rate of 1000 Hz and online low-pass filtered at 200 Hz. To ensure a good signal-to-noise ratio, electrode impedance was kept below 50kΩ, as recommended by the manufacturer. Additionally, an online Cz reference montage was used, including CPz as the ground electrode.

The original data recording was cleaned and processed using the EEGLAB open-source toolbox^39^ for MATLAB (2023b, Mathworks Inc, Natick, USA). For the preprocessing, the data were initially downsampled to 500 Hz and cleaned for frequency-specific noise artifacts using the adaptive automatic removal toolbox Zapline-Plus.^40^ Afterwards, the data were filtered through a bandpass filter set between 3 and 30 Hz. The clean_rawdata function^41,42^ was then utilised to eliminate channels affected by transient or high-amplitude noise (line noise threshold: 4), channels with low correlation to neighbouring channels (correlation threshold: 0.8), and channels exhibiting prolonged flatline activity (flatline threshold: 5). Additionally, automatic subspace reconstruction was applied to remove and interpolate non-stationary high-amplitude bursts with significant variance (burst threshold: 10). Subsequently, the Cz reference channel was restored, the data were re-referenced to common average and down-sampled to 250 Hz. The scalp signals were decomposed into independent components (ICs) using adaptive mixture independent component analysis.^43^ This dataset comprised roughly 90,000 data points (equivalent to 6 min of recording at a 250 Hz sampling rate) and facilitated the decomposition of signals from only clean EEG channels. The ICLabel classifier^44^ was employed to distinguish and reject non-neural ICs (Muscle 70%, Eye 70%, Heart, Line Noise 90%, Channel Noise 90% and Other 90% probability), ensuring only those with the highest likelihood of neural origin were retained. Finally, rejected channels were interpolated to maintain a consistent head montage across participants.

### EEG graph analysis

**Fig. 2:**
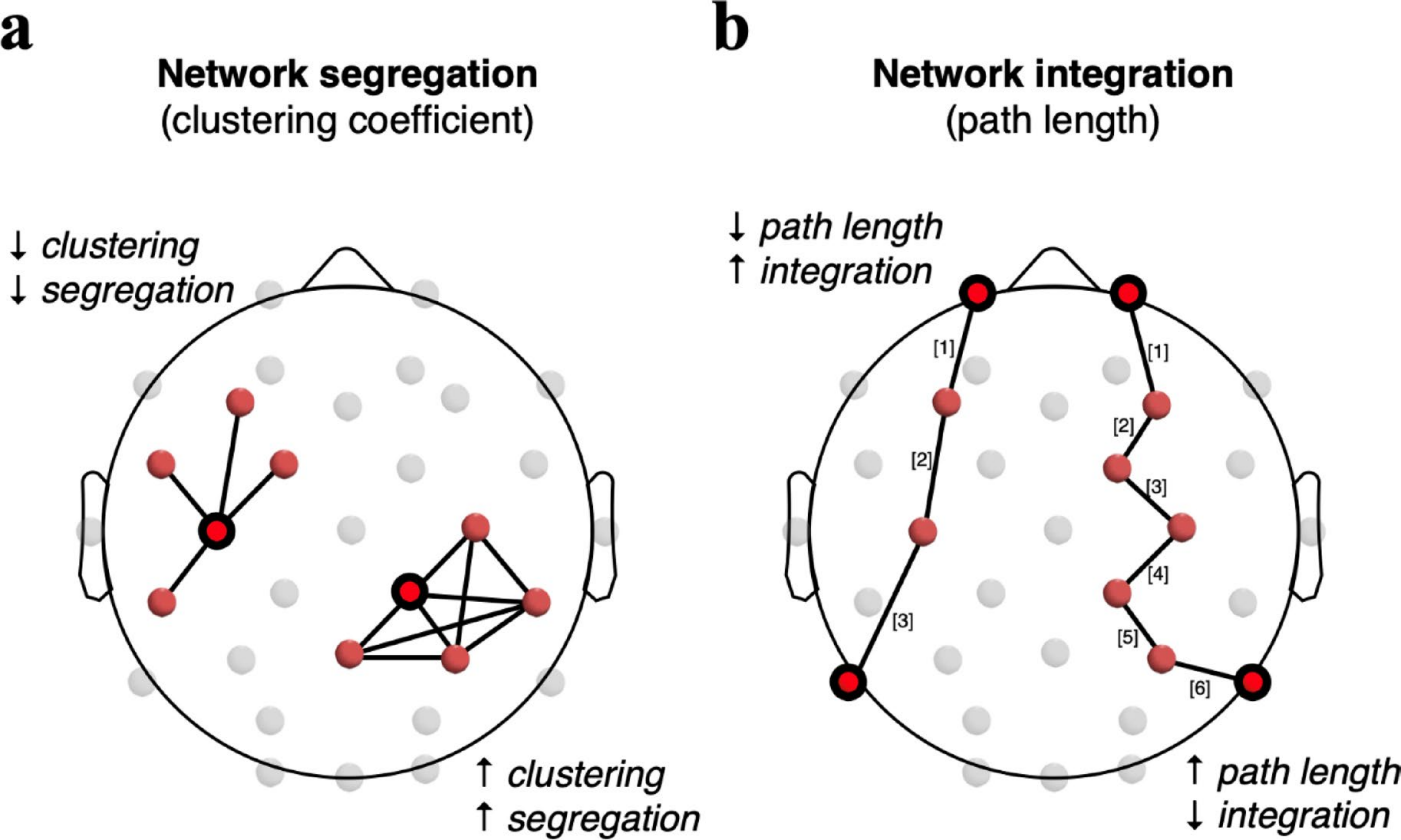
Illustration of the graph network metrics. a) Clustering coefficient as a measure of network segregation, representing the tendency of nodes to form local clusters / triangles. Path length as measure of network integration, representing the average smallest number of edges (steps) that need to be travelled to move from one node to another.

For the computation of weighted, undirected brain graphs,^45,46^ functional connectivity matrices were first generated for each participant and condition, as well as theta (4–7 Hz), alpha-1 (8–10 Hz), and alpha-2 (10–12 Hz) frequency bands using the FCLAB toolbox.^47^ The phase lag index (PLI)^48^ was employed as a metric of dynamic phase coupling, estimating functional connectivity by quantifying statistical dependencies between pairs of EEG channels. The resulting adjacency matrices were then processed using the functions of the Brain Connectivity Toolbox.^49^ First, real PLI values were normalised according to the rank of each participant’s maximum and minimum values. Then, the complexity of the graphs was reduced by applying an arbitrary thresholding, only keeping the strongest 30% of edges. Finally, graph measures were calculated as key outcomes of interest (Fig. 2). The clustering coefficient (CC), as a measure of network segregation, indicates the tendency of a network to form local triangles within its immediate vicinity and was computed as follows:

$$\:C=\frac{1}{n}\sum\:_{i\in\:N}{C}_{i}=\frac{1}{n}\:\sum\:_{i\in\:N}\frac{{2t}_{i}}{{k}_{i}({k}_{i}-1)}$$

where *C*_*i*_ is the clustering coefficient of node *i*.^49^

The path length (PL), as a measure of network integration, quantifies the average number of steps required for information to travel from the node of interest to any other node of the network. It was computed as follows:

$$\:L=\frac{1}{n}{\sum\:}_{i\in\:N}{L}_{i}=\frac{1}{n}\:{\sum\:}_{i\in\:N}\frac{{\sum\:}_{j\in\:N\:j\ne\:i}{d}_{ij}}{n-1}$$

where L_i_ denotes the mean distance from node i to all other nodes in the network.^49^.

As final outcomes, global CC and global PL were calculated as the mean values across all nodes. Thus, the combination of CC and PL was deemed to provide valuable insights into the underlying functional architecture of the brain related to postural control processes during eyes-open and -closed single-leg standing.

### Statistical analysis

Descriptive statistics were calculated for the main outcome variables and presented as means ± standard deviations, as well as boxplots displaying medians and interquartile ranges for each group and condition. For all comparisons, the ACLR injured leg value was calculated as the mean of three trials for that leg. In the control group, data from both dominant and non-dominant legs were pooled, and outcome values were calculated as the mean across all six trials. This was done based on a recent recommendation^50^ which indicates that, particularly for one-leg balance tasks, balance performance does not seem to be influenced by limb dominance, and thus, both legs can be used as a reference. Group differences between ACLR and control participants, along with potential sex differences, were analysed using a permutation-based two-way analysis of covariance (ANCOVA),^51^ implemented in R (version 4.1.1.),^52^ using the *permuco* package,^51^ to provide valid and robust inference in the presence of covariates without relying on strict distributional assumptions of normality. Statistical significance was assessed using the Freedman–Lane permutation scheme.^53^ Separate models were fitted for eyes-open and eyes-closed conditions, with the dependent variable being the respective outcome measure (postural measures, EEG graph measures, or knee flexion) for each condition. In the between-participant models, the independent variables were group (ACLR vs. control) and sex. Relative double support time was included as a covariate for all outcome measures to account for individual variations. As this variable was zero for all participants in the eyes-open condition, it did not contribute to those models. In addition, paired permutation tests^51^ were conducted within the ACLR group to compare balance performance between the injured and non-injured leg. In these repeated-measures models, the dependent variable was the respective outcome measure, with leg (injured vs. non-injured) as the independent variable of interest, testing the effect of injury, and sex included as a covariate. Statistical significance for these repeated-measures analyses was assessed using the Kherad-Pajouh and Renaud permutation scheme.^54^ For all comparisons, effect-size estimates were assessed using ηp^2^.^55^ Separate models were fitted for the eyes-open and eyes-closed conditions, with relative double support time included as a covariate to control for individual variations in postural control. Statistical inference for all tests was performed using 50,000 permutations. P-values were calculated to assess the main effects of group and sex, as well as the effect of injury (injured vs. non-injured leg) within the ACLR group on postural and EEG graph measures, as well as knee flexion. Significance level was set to *p* < 0.05. To control for multiple comparisons, the false discovery rate correction of Benjamini and Hochberg^56^ was applied separately to postural and EEG-derived measures, and to group, sex, and leg comparisons across conditions.

## Results

Descriptive statistics for all outcome variables are presented in Table 2.

### EEG graph measures

**Fig. 3:**
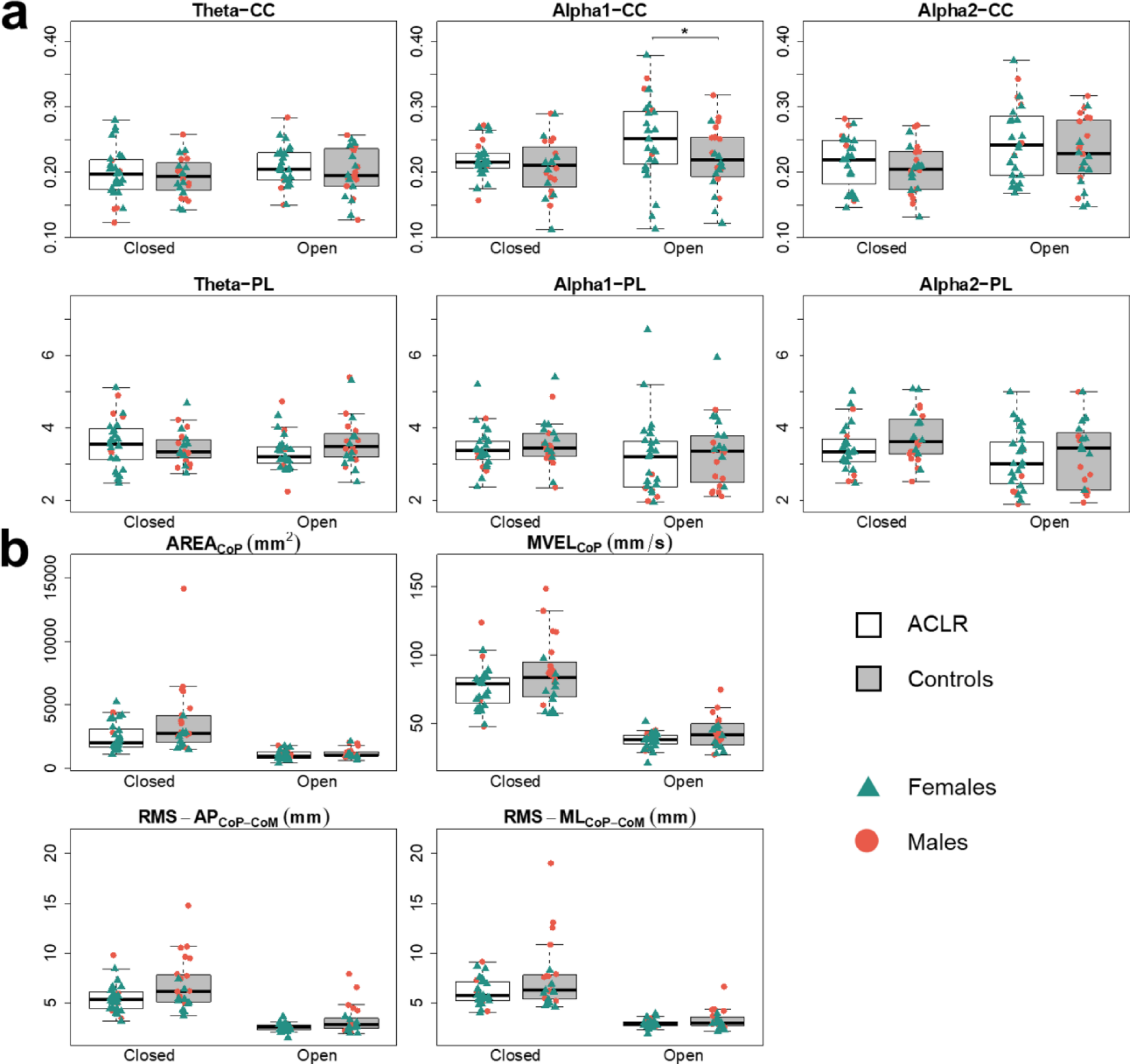
Main results, presented for both eyes-open and eyes-closed conditions. Data are presented on a group basis and with an indication of the participants’ sex. Significant group differences are indicated. (**a**) Graph theoretical measures, derived from electroencephalography data. For each frequency band, we calculated the Clustering coefficient (CC) as a measure of network segregation (arbitrary units, 0–1) and Path length (PL) as a measure of network integration (average number of edges). (**b**) Posturographic measures, calculated based on centre of pressure (CoP) and based on the mean differences between CoP and the centre of Mass (CoM) ACLR, Anterior Cruciate Ligament Reconstruction (group); CC, Clustering Coefficient; PL, Path Length; CoP, centre of preseure; CoM; centre of mass; AREA_CoP_, 95% ellipse area around the CoP sway centre; MVEL_CoP_, Mean CoP Velocity; RMS, Root Mean Square; AP, Anterior-Posterior direction; ML, Medial-Lateral direction.

Group differences in brain functional connectivity were observed exclusively in the eyes-open condition. Compared to controls, the ACLR group exhibited higher CC (*F(1*,*48) = 8.81*, *p* = 0.025,* ηp² = 0.155*) in the alpha-1 frequency band (Fig. 3a, indicative of increased network segregation). No other group differences were observed.

### Biomechanical outcome measures

Across both groups, touchdowns occurred only during the eyes-closed condition. In total, 13 participants (48%) in the ACLR group and 16 participants (67%) in the control group experienced at least one touchdown across trials. However, touchdowns were minimal relative to the 30-s trials. Expressed as relative double-support time, values were 1.1 ± 2.7% (injured leg), 1.2 ± 2.9% (non-injured leg), and 2.0 ± 3.7% (controls, pooled), with no significant between-group differences. After accounting for sex and touchdown in the model, no significant group differences were found for any of the sway characteristics (Fig. 3b; Table 2). Regarding mean knee flexion angle, an overall greater flexion was observed during eyes-closed trials for both groups, compared to eyes-open trials. A significant between-leg effect was observed during eyes-open trials (F(1,26) = 4.86, *p* = 0.036, ηp² = 0.157), with greater knee flexion angle seen for the ACLR leg (7.4^◦^ ± 5.2^◦^) compared to the contralateral leg (5.5^◦^ ± 6.6^◦^).

### Sex-related differences

Sex-related differences in brain functional connectivity were identified in the eyes-open condition, independent of group allocation. Specifically, males exhibited higher clustering coefficient and shorter path length in both alpha-1 (F(1,48) = 9.20, *p* = 0.007 ηp² = 0.161; F(1,48) = 12.38, *p* = 0.003, ηp² = 0.205 for CC and PL, respectively) and alpha-2 (F(1,48) = 8.93, *p* = 0.007, ηp² = 0.157; F(1,48) = 11.69, *p* = 0.004, ηp² = 0.196 for CC and PL, respectively) frequency bands, indicating a more segregated and integrated network. Behaviourally, sex-related differences were observed in both eyes-open and eyes-closed conditions, with males demonstrating higher postural sway measures compared to females. For eyes-open trials, males demonstrated greater AREA_CoP_ (F(1,48) = 5.76, *p* = 0.042, ηp² = 0.107), MVEL_CoP_ (F(1,48) = 4.55, *p* = 0.049, ηp² = 0.087), and RMS-AP_CoP−CoM_ (F(1,46) = 7.16, *p* = 0.031, ηp² = 0.135) compared to females. For eyes-closed trials, males demonstrated greater AREA_CoP_ (F(1,47) = 7.94, *p* = 0.007, ηp² = 0.144), MVEL_CoP_ (F(1,47) = 11.16, *p* = 0.003, ηp² = 0.192), RMS-AP_CoP−CoM_ (F(1,46) = 11.75, *p* = 0.003, ηp² = 0.204) and RMS-ML_CoP−CoM_ (F(1,46) = 8.57, *p* = 0.007, ηp² = 0.157) compared to females. All the sex-related differences are illustrated in Fig. S1 in Supplementary material.

**Table 2:** Main outcome variables (Mean ± SD) across groups and conditions. Significant findings (Benjamini-Hochberg adjustment) are highlighted.

			ACLR (Injured Leg)	ACLR (Non-injured Leg)	Controls (Pooled Legs)
Brain graph metrics					
Open	Theta	CC (a.u.)	0.2 ± 0.0	0.2 ± 0.0	0.2 ± 0.0
		PL (N steps)	3.3 ± 0.5	3.5 ± 0.7	3.6 ± 0.7
	Alpha-1	CC ^a, c^	0.3 ± 0.1	0.2 ± 0.1	0.2 ± 0.0
		PL ^c^	3.2 ± 1.0	3.3 ± 1.3	3.3 ± 0.9
	Alpha-2	CC ^c^	0.2 ± 0.1	0.3 ± 0.1	0.2 ± 0.0
		PL ^c^	3.1 ± 0.8	3.1 ± 0.8	3.3 ± 0.9
Closed	Theta	CC	0.2 ± 0.0	0.2 ± 0.0	0.2 ± 0.0
		PL	3.6 ± 0.7	3.5 ± 0.5	3.4 ± 0.4
	Alpha-1	CC	0.2 ± 0.0	0.2 ± 0.0	0.2 ± 0.0
		PL	3.4 ± 0.6	3.4 ± 0.7	3.6 ± 0.6
	Alpha-2	CC	0.2 ± 0.0	0.2 ± 0.0	0.2 ± 0.0
		PL	3.4 ± 0.6	3.3 ± 0.6	3.7 ± 0.7
Behavioural metrics					
Open	CoP	AREA (mm²) ^c^	1038.1 ± 341.1	968.5 ± 339.4	1155.1 ± 402.2
		MVEL (mm/s) ^c^	37.6 ± 5.9	38.6 ± 6.1	43.1 ± 11.2
	CoP-CoM	RMS-AP (mm) ^c^	2.6 ± 0.4	2.6 ± 0.5	3.3 ± 1.4
		RMS-ML (mm)	3.0 ± 0.4	3.0 ± 0.4	3.3 ± 0.9
	Knee Flexion		7.4 ± 5.2	5.5 ± 6.6	4.2 ± 5.7
Closed	CoP	AREA (mm²) ^c^	2722.7 ± 1682.1	2817.1 ± 1762.4	3611.8 ± 2633.3
		MVEL (mm/s) ^c^	77.5 ± 17.5	80.9 ± 20.7	86.2 ± 23.2
	CoP-CoM	RMS-AP (mm) ^c^	5.8 ± 2.0	6.5 ± 3.0	6.9 ± 2.6
		RMS-ML (mm) ^c^	6.8 ± 3.2	6.6 ± 2.1	7.6 ± 3.3
	Knee Flexion ^b^		10.9 ± 4.9	9.4 ± 5.2	10.4 ± 7.4

^a^: Significance between groups.

^b^: Significant between ACLR legs.

^a^: Significant between sexes.

ACLR, Anterior Cruciate Ligament Reconstruction; a.u., arbitrary units; CC, Clustering Coefficient; PL, Path Length; CoP, centre of pressure; CoM, centre of mass; AREA, 95% ellipse area around the CoP sway centre; MVEL, Mean CoP Velocity; RMS, Root Mean Square; AP, antero-posterior; ML, medio-lateral.

## Discussion

The primary objective of this study was to examine variations in functional brain connectivity following ACLR during one-leg static balance tasks, particularly in relation to the presence or absence of visual input. The present findings revealed that only in the eyes-open condition, individuals after ACLR demonstrated enhanced cortical network segregation in the alpha-1 frequency band, when compared to their uninjured counterparts. This, however, was not accompanied by behavioural group differences in any of the posturographic measures. In the eyes-open condition, ACLR group demonstrated a kinematic leg-difference with greater mean knee flexion angles in the ACLR leg, compared to their contralateral leg. Additionally, our study yielded an incidental finding regarding sex-related differences in both functional connectivity and behavioural measures. Specifically, independent of group, males demonstrated greater clustering coefficient and shorter path length in both alpha-1 and alpha-2 frequencies during eyes-open trials, and larger CoP sway area, sway velocity, and greater CoP-CoM distance in both conditions, compared to females.

### Functional brain connectivity

In the present study, features of network integration and segregation were deemed to provide insights into the underlying functional architecture of cortical processes associated with eyes-open and eyes-closed single-leg standing. As such, individuals following ACLR showed significantly stronger network segregation in the alpha-1 frequency band during single-leg standing when visual information is available, indicating that specialised regions or sub-networks tended to work independently.^24^ In the present study, group differences in network segregation were only observed in the alpha‑1 band, but not in the theta band. Alpha activity has frequently been linked to functional inhibition, sensory gating and the regulation of information flow within large-scale sensorimotor networks in eyes-open conditions.^57^ In contrast, theta-band activity is more commonly associated with cognitive control, memory-related processes and task-related demands.^58^ The presence of group differences specifically in the alpha‑1 band may therefore suggest that alterations in intrinsic cortical network organization after ACLR might more closely relate to mechanisms involved in sensory integration and the modulation of ongoing cortical communication, rather than processes typically reflected in theta-band dynamics.

In archetypical functional brain networks, optimal segregation allows for specialised processing within distinct areas of the brain.^59^ Interestingly, this network characteristic of higher segregation was observed in the ACLR group compared to the non-injured controls during the eyes-open single-leg stance, despite similar postural sway characteristics for both groups. Although graph-theoretical analyses of postural control mechanisms remain scarce, Liang and colleagues^60^ recently observed in young adults that greater balancing difficulty was associated with segregated information processing, as reflected by an increased CC. In line with this, the current data also indicated that increased balance difficulty might be accompanied with greater segregation in cortical processing, in males compared to females. Furthermore, Varghese et al.^61^ observed that in response to increased demands for maintaining postural equilibrium, adjacent cortical regions establish short-range neural connections to support the necessary integrative processes for balance regulation. These observations support the notion that, based on our findings in individuals with ACLR, the brain may increase local information processing within small subnetworks when vision is available as a result of injury-related balance challenges. In contrast, populations with actual damage to brain structures, such as patients with mild traumatic brain injury, were shown to demonstrate less segregation than asymptomatic controls, presumably indicative of lower neural efficiency.^26^ Studies utilising brain imaging^62,63^ further show that, in the early stages of learning, motor and visual systems are closely integrated while as learning advances, these systems become more autonomous, each engaging in independent processing characterised by distinct temporal patterns of brain activity and connectivity. Consequently, when visual information is available, individuals after ACLR may have learned to integrate this input into neural circuits to a greater extent, particularly in response to the elevated postural demands due to injury-related sensorimotor alterations.^13,64–67^ Conversely, when visual input is absent, individuals after ACLR are forced to rely on consolidated and automatised processing pathways based on proprioceptive and vestibular inputs, leading to a reset of compensatory strategies and postural control mechanisms similar to those of non-injured individuals under the same condition.

Further investigation of cortical network segregation and integration based on graph theory may provide a valuable perspective for a better understanding of compensatory cortical mechanisms in individuals after ACLR. By leveraging advanced graph-based analyses, it appears possible to quantify the degree to which the brain functionally reorganises following injury, identifying the neural underpinnings of compensation strategies in injured populations.

### Behavioural measures

Although the ACLR group reported functional deficits in e.g., sport & recreation and quality of life, they did not exhibit any behavioural deficiencies in postural control in neither eyes-open nor eyes-closed trials. This was regardless of measures based on CoP or CoP-CoM. This is in line with a previous investigation utilising 30s eyes-open single leg stance with 414 individuals after ACLR,^68^ who reported non-impaired balance of the injured limb. Our CoP-based lack of differences were also somewhat expected given previous work by Lehmann et al.,^23^ who tested individuals subacutely following ACLR in a comparable task, and reported no deficiencies in postural control, assessed primarily by CoP sway area and velocity. Given the nature of the measures, it is likely that larger CoP sway area and velocity typically indicate more excessive instability, which would require a higher level of postural disruption to show significant differences. It has previously been shown that compared to both CoP and CoM in isolation, the CoP-CoM measure displays a significantly higher mean power frequency characteristic.^69^ In other words, it fluctuates to a much greater extent than the CoP alone, making it a more sensitive measure for postural adjustments during quiet standing. Moreover, although the CoP can be viewed as the net neuromuscular response controlling displacement of the CoM,^38^ the CoP-CoM distance is reflective of the error signal that causes the CoM to horizontally accelerate,^69^ making it a more sensitive measure of neuromuscular coordination. The CoP-CoM distance can therefore be expected to detect subtle variations in postural control, even in the absence of large CoP displacements. Regardless, we did not observe any behavioural sway differences at group level for any of the current conditions. Interestingly, despite no balance deficiencies, the ACLR group did display greater mean knee flexion in their injured leg compared to the contralateral leg during eyes-open trials. It is possible that individuals following ACLR have employed a compensatory strategy to maintain balance, lowering their CoM by flexing their knee. This strategy, seen during eyes-closed regardless of group, may have resulted in normative CoP and CoP-CoM sway characteristics, although likely requiring constant muscular co-contraction. This adaptation may reflect high-level neural mechanisms,^70^ manifested as compensatory kinematics, although at a possible metabolic cost of constant muscular activation required during prolonged one-leg stance with a flexed knee.

No group differences were observed in eyes-closed trials. Over-reliance on visual input to compensate for impaired proprioception following ACL-injury has been extensively discussed.^13–15,21,71^ Balancing with eyes-closed is a naturally more challenging condition, as indicated by significantly greater sway area and velocity for both groups compared with the eyes-open condition. Our findings indicate that the impact of ACL-injury on condition-dependent loss of stability is non-significant. These results align with Miko et al.,^72^ whose comparison included both eyes-closed and dual-task conditions. They found that any group differences in postural control were driven by the cognitive dual-task and were not apparent during single-task with eyes-closed. This implies that detecting group differences may require increased task complexity, specifically, a task demanding more divided attention, which reduces the relative automaticity of maintaining balance.^73,74^ Notably, merely increasing task difficulty (e.g., removing vision) is likely to reduce motor automaticity and make maintaining balance a more conscious effort.^75,76^ In the eyes-closed condition, and regardless of group, participants in our study had repeated instances of touchdowns resulting in greater sway characteristics for both groups. It is also possible that these large displacements (albeit controlling for touchdowns in the statistical models) clouded any ACLR-related differences, even based on assessing a relatively sensitive CoP-CoM characteristic. Interestingly, in Miko et al.,^72^ the eyes-closed condition task was presumably less physically-demanding than in our study, given no information being reported regarding touchdowns. This was possibly due to considerably shorter 20-second trials in their study, whereas our study required participants to balance with their eyes closed for 50% longer, substantially increasing the difficulty. Still, the absence of group differences in both Miko et al. and our study suggests that removing vision may already increase attentional demands for both ACLR and control participants, and rather than being merely a physical challenge, a cognitive component may be the key missing element after ACLR.^72–74^ Furthermore, with no neural or behavioural group differences shown during eyes closed, it is likely that the complete lack of visual input forces the utilisation of proprioceptive feedback regardless of an ACL injury background. Conversely, greater cortical segregation and kinematic adaptations in the ACLR leg were pronounced when visual information was available, supporting the notion of sensory reweighting^77^ during eyes-open, due to post-injury neuroplasticity.^13,14^ This has implications for real-life situations – in which eyes are usually open – during which post-ACLR adaptations might compensate for potential deficiencies, possibly resulting in muscular fatigue.

### Methodological considerations for EEG-derived brain graphs

Our selected approach of leveraging connectivity matrices and graph analysis provides a detailed structural description allowing for a comparison of diverse network topologies within a robust theoretical framework. Thereby, the analysis not only underscores interactions between directly connected brain areas but also captures functional interactions without direct anatomical links.^24^.

Despite the promising insights by using sensor-based functional connectivity for graph theoretical analysis, several methodological considerations should be acknowledged. First, the absence of source-level analysis may have limited the anatomical specificity of the observed connectivity patterns. Several studies suggested that connectivity estimates might be more accurate when derived from reconstructed cortical sources, rather than scalp-level signals.^78^ However, mobile EEG studies pose unique challenges for source modelling, including increased movement-related artifacts, reduced electrode density and invariant sources identified across participants, which may compromise the accuracy of inverse solutions and statistical analyses.^79^ Given these constraints, sensor-level connectivity analysis still provides a feasible and robust alternative that balances methodological rigor with ecological validity in mobile experimental settings.

A second consideration regarding connectivity estimates derived from EEG sensor-level data in the present study concerns the issue of volume conduction and source leakage. EEG electrodes record linearly mixed signals from multiple cortical and non-cortical sources, which can result in spurious zero-lag connectivity due to shared source activity rather than true functional interactions.^80^ While the PLI was explicitly developed to minimise zero-phase lag interactions and reduce sensitivity to volume conduction effects,^48,81^ residual contamination from common sources cannot be fully ruled out. Consequently, spatial leakage may ultimately artificially inflate the connectivity measures and potentially lead to spurious interpretations of network topology.^82^ In the present study, we therefore utilised ICA-based approaches to reduce the amount of spurious connectivity by decomposing the mixed signals recorded at the scalp into statistically independent components and identifying artifact signals such as eye blinks, muscle activity, cardiac signals or electrode movement.^41,83^ By isolating these non-neural sources and selectively removing artifact components without discarding neural data, the signal-to-noise ratio and the quality of the functional connectivity estimates were substantially improved. Furthermore, removing artifacts at the component level preserves more genuine brain activity compared to traditional methods like channel rejection or excessive filtering, which can result in data loss or distortion.^84^.

Lastly, although CC and path PL are among the most frequently used graph theoretical metrics for quantifying functional segregation and integration of brain networks, their interpretation is highly sensitive to methodological parameters.^85^ Key factors include the selection of the connectivity measure, thresholding strategies for adjacency matrices, and the number and definition of nodes in the network.^86^ Small changes in these parameters can substantially alter the resulting network topology, potentially biasing CC and PL estimates. For instance, arbitrary thresholding can artificially inflate or suppress network density, leading to misleading conclusions regarding the balance between local clustering and global efficiency.^87^ In the present study, a proportional threshold was used to keep network density constant across participants/conditions,^86^ yet we acknowledge that graph metrics can be threshold-dependent.^49^ Future studies should therefore evaluate the robustness of the findings across multiple proportional thresholds (or use threshold-free approaches such as weighted graphs or area-under-the-curve across densities) to confirm that the present observations are not dependent on a single density value. Furthermore, the choice of connectivity metric – such as the PLI – influences the weights and directionality of edges, which in turn impacts graph measures. In mobile EEG studies, additional factors such as increased movement artifacts and non-stationarity of data can further affect network stability and the reliability of CC and PL.^45,88^ Despite these limitations, we maintained methodological consistency and appropriate controls were applied, making CC and PL valuable descriptors of network organisation, which offer meaningful insights into brain functional connectivity.^25^.

### Methodological considerations (for the study sample, protocol and behavioural measures)

Several aspects of the study sample and protocol need to be considered when interpreting the present findings. Firstly, while we considered sex as a confounding variable to mitigate its influence, it is possible that sex-related differences in postural control^68^ and knee functionality^89^ after ACLR may still have impacted our results due to the imbalanced number of females and males in the two groups. Notably, if sex was a key factor, given the fact that females had lower segregation compared to males we would have expected lower segregation in the ACLR group, in which females represented the majority. Instead, we observed the opposite, further indicating that group differences were not influenced by the sex imbalance factor. Nevertheless, future research with a balanced proportion of female and male participants is recommended. Secondly, pooling EEG and behavioural data in the control group may have limited the interpretation of our results. While previous studies indicate differences in graph analysis related to upper limb dominance,^90^ we conducted a separate analysis confirming no significant differences in graph parameters between dominant and non-dominant lower limbs in either group, thus supporting the choice of pooling data from both limbs in the controls. We further posit that given the lack of leg differences for the controls, choosing either the dominant or non-dominant leg for comparisons with the ACLR leg was a question of preference and would thus have introduced a certain bias. Pooling the legs thus seemed the more rigorous choice to handle the group comparisons. Still, given that some individuals in the ACLR group had injured their dominant leg while others injured their non-dominant leg, the matching between injured and dominant leg could have confounded the results. To explored this, we performed a supplementary a subgroup analysis. Although neither subgroup differed significantly from the controls, differences between the ACLR subgroups were observed in the eyes-open condition, with relatively worse balance performance in those who had injured their non-dominant leg (*Supplementary material*). Previous work by Calisti et al.^91^ reported ACLR differences depending on injured leg dominance, though in contrast to our findings, greater deficits were observed following dominant-leg injuries. Notably, their study assessed dynamic postural control during landing. In dynamic contexts, the dominant leg is typically the kicking leg, whereas the non-dominant leg often serves a stabilizing role, which may explain why dominant-leg injuries resulted in greater deficits. However, during quiet unipedal stance without an external perturbation or goal-directed movement, it is possible that the dominant leg would perform relatively better. According to a systematic review and meta-analysis by Kiss et al.,^92^ (correlations between different types of balance performance (static, dynamic, proactive and reactive) are generally small, indicating that balance is largely task-specific. Thus, findings from dynamic balance assessments may not directly generalise to static balance tasks like those used in the present study. Given the small subgroup sizes, these results should however be interpreted with caution. Future work should further explore the interaction between injury side, leg dominance and balance performance in different contexts.

Thirdly, ACLR participants had a wide range in time since surgery, some still undergoing rehabilitation (*N* = 11), which might have influenced brain or balance measures. To address this, we performed confirmatory correlation analyses between time post-surgery and all outcomes (*Supplementary material*). None of the behavioural correlations were significant, and all coefficients were negligible. Some EEG variables in the eyes-closed condition showed marginal significance but given the large number of comparisons and the marginal p-values, these likely represent type I errors. We also performed a subgroup analysis comparing participants who had completed rehabilitation with those who had not, and did not find significant differences for any of the outcome variables. Thus, we remain confident that neither time since surgery nor rehabilitation completion affected functional brain connectivity or balance outcomes. Fourthly, although unlikely given that these were generally minor, concomitant meniscal or ligamentous injuries in the ACLR group were not accounted for and may introduce some heterogeneity, potentially influencing the outcomes. Finally, while allowing touchdowns enhanced the ecological validity of the balance trials, it could have introduced a potential confound. Although we accounted for this in the statistical models, our findings should still be interpreted with caution.

## Conclusions

The present study sought to investigate characteristics of functional brain networks associated with postural control in individuals after ACLR, specifically focussing on the effect of visual information on static one-leg standing and cortical processing. Individuals post-ACLR exhibited heightened segregation of neural processing during unilateral balance tasks on their injured leg, exclusively under visual input conditions, despite similar sway characteristics as the control group. This was accompanied by greater knee flexion observed in their injured leg. Collectively, these findings suggest compensatory neural adaptations during visually guided regulation of postural equilibrium, potentially aiding stability. Future work should expand on these notions by incorporating similar approaches during various and more complex functional tasks. Additionally, clinicians and sport practitioners are advised to be aware of potential movement adaptations, particularly when guiding athletes back to their respective sport following ACL injury.

## Electronic Supplementary Material

Below is the link to the electronic supplementary material.

Supplementary Material 1

## Data Availability

The datasets generated during and/or analysed during the current study are available from the corresponding author on reasonable request.
