# Correction: Visual information modulates brain network characteristics during static balance following ACL reconstruction – A graph theoretical analysis

**DOI:** 10.1038/s41598-026-56238-6

**Published:** 2026-06-02

**Authors:** Adam Grinberg, Tim Lehmann, Johan Strandberg, Gjergji Cobani, Charlotte K. Häger

**Affiliations:** 1 Department of Community Medicine and Rehabilitation, Umeå University, Umeå, Sweden; 2 Exercise Science & Neuroscience Unit, Department of Exercise & Health, Faculty of Science, Paderborn University, Paderborn, Germany; 3 Department of Statistics, Umeå University, Umeå, Sweden; 4 Department of Diagnostics and Intervention, Umeå University, Umeå, Sweden; 5 Clinics of Orthopaedics, University Hospital of Umeå, Umeå, Sweden

Correction to: *Scientific Reports* 10.1038/s41598-026-52086-6, published online 06 May 2026

The original version of this Article contained errors in Table 1, where the values for “Symptoms” and “Pain” in the column “Controls (n=24)” were incorrect. The correct and incorrect values appear below.

Incorrect:

**Table:** 

	**Controls (n=24)**
Symptoms	97.6 (89.3–100.0)
Pain	100.0 (97.9–100.0)

Correct:

**Table:** 

	**Controls (n=24)**
Symptoms	96.4 (89.3–100.0)
Pain	100.0 (100.0–100.0)

The original Article has been corrected.

