# Supplementary material for: Visual information modulates brain network characteristics during static balance following ACL reconstruction – A graph theoretical analysis
Source: Sci Rep. 2026 May 6;16:14430. doi: 10.1038/s41598-026-52086-6 (PMC13149860; doi:10.1038/s41598-026-52086-6)
Supplement: Supplementary file 1 — Supplementary Material 1 [file 41598_2026_52086_MOESM1_ESM.docx]

**Supplementary Material – Sex differences**

**
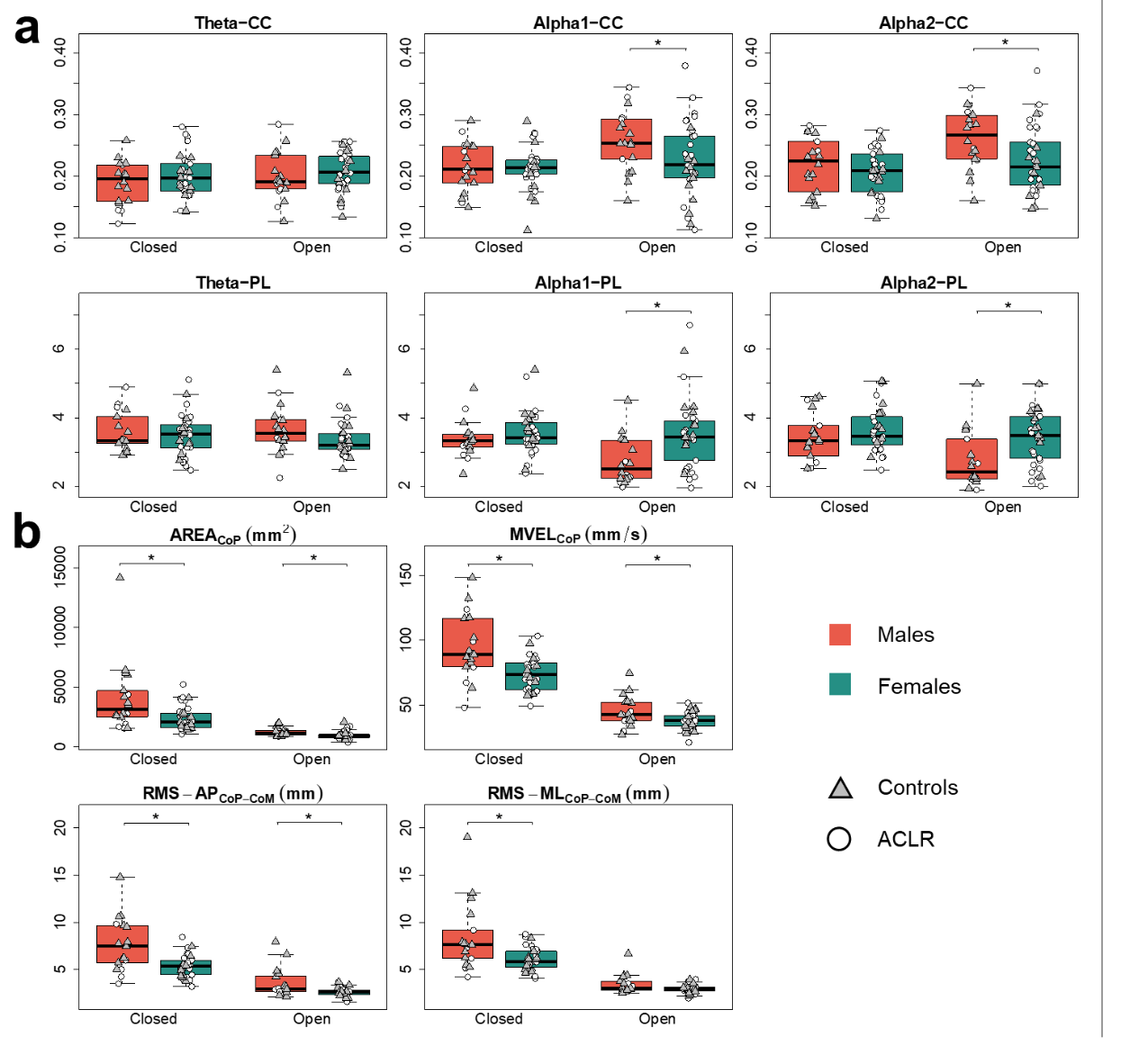
**

**Fig. S-1., Sex-related differences in all the dependent variables (incidental finding), presented for both eyes-open and eyes-closed conditions.** Data are presented on a sex basis and with an indication of the participant’s allocation to either ACLR or control group. Significant sex differences are indicated. A) Graph theoretical measures, derived from electroencephalography data. For each frequency band, we calculated the Clustering coefficient as a measure of network segregation (arbitrary units, 0-1) and Path length as a measure of network integration (average number of edges). B) Posturographic measures, calculated based on centre of pressure (CoP) and based on the mean differences between CoP and the centre of mass (CoM).

ACLR, Anterior Cruciate Ligament Reconstruction (group); CC, Clustering Coefficient; PL, Path Length; CoP, centre of preseure; CoM; centre of mass; AREA_CoP_, 95% ellipse area around the CoP sway centre; MVEL_CoP_, Mean CoP Velocity; RMS, Root Mean Square; AP, Antero-posterior direction; ML, Medio-lateral direction.

**Supplementary Material – Time post-surgery correlations**

Behavioural measures

I – Injured leg; NI – Non-injured leg

EEG-derived measures

I – Injured leg; NI – Non-injured leg

**Supplementary Material – Subgroup analysis (injury-dominance matching)**

Results of permutation-based ANCOVAs for dominancy (injured dominant leg vs injured non-dominant leg), accounting for sex and relative double support time as a covariate (in eyes closed condition), across study outcome measures. F-values (df_1_, df_2_), Benjamini-Hochberg–adjusted p-values, and partial $\eta_{p}^{2}$ are reported, with significant values shaded and highlighted in bold.

| **Outcome measure** | | | **ACLR Subgroup (F; p;** $\boldsymbol{\eta}_{\boldsymbol{p}}^{\boldsymbol{2}}$**)** | | **Sex (F; p;** $\boldsymbol{\eta}_{\boldsymbol{p}}^{\boldsymbol{2}}$**)** | |
| --- | --- | --- | --- | --- | --- | --- |
|  |  |  | Open | Closed | Open | Closed |
| Brain graphmetrics | Theta | CC | 0.222 (1,24);  0.971; 0.009 | 0.349 (1,23);  0.668; 0.015 | 1.090 (1,24);  0.369; 0.043 | 4.724 (1,23);  0.210; 0.170 |
|  |  | PL | 0.051 (1,24);  0.991; 0.002 | 0.708 (1,23);  0.613; 0.030 | 0.437 (1,24);  0.517; 0.018 | 3.582 (1,23);  0.210; 0.135 |
|  | Alpha-1 | CC | 1.526 (1,24);  0.971; 0.060 | 1.662 (1,23);  0.422; 0.067 | 3.736 (1,24);  0.112; 0.135 | 0.172 (1,23);  0.816; 0.007 |
|  |  | PL | 0.686 (1,24);  0.971; 0.028 | 2.294 (1,23);  0.422; 0.091 | 4.913 (1,24);  0.112; 0.170 | 0.008 (1,23);  0.931; <0.001 |
|  | Alpha-2 | CC | 0.407 (1,24);  0.971; 0.017 | 0.007 (1,23);  0.933; <0.001 | 3.415 (1,24);  0.112; 0.125 | 2.604 (1,23);  0.245; 0.102 |
|  |  | PL | <0.001 (1,24);  0.991; <0.001 | 2.047 (1,23);  0.422; 0.082 | 3.955 (1,24);  0.112; 0.141 | 0.505 (1,23);  0.730; 0.022 |
| Behavioural outcome-measures | CoP | AREA | 0.442 (1,24);  0.511; 0.018 | 1.815 (1,23);  0.381; 0.073 | 3.003 (1,24);  0.378; 0.111 | 0.239 (1,23); 0.642; 0.010 |
|  |  | MVEL | **9.016 (1,24);**  **0.019; 0.273** | 0.668 (1,23);  0.424; 0.028 | 0.314 (1,24);  0.640; 0.013 | 1.305 (1,23);  0.642; 0.054 |
|  | CoP-CoM | RMS-AP | **6.628 (1,23);**  **0.022; 0.224** | 0.689 (1,23);  0.424; 0.029 | 1.640 (1,23);  0.430; 0.067 | 0.241 (1,23);  0.642; 0.010 |
|  |  | RMS-ML | **6.477 (1,23);**  **0.022; 0.220** | 4.853 (1,23);  0.152; 0.174 | 0.224 (1,23);  0.640; 0.010 | 0.344 (1,23);  0.642; 0.015 |
|  | Knee flexion | | 2.513 (1,24);  0.128; 0.095 | 0.398 (1,23);  0.530; 0.017 | 0.740 (1,24);  0.400; 0.030 | <0.001 (1,23);  0.989; <0.001 |
